# Distribution of pathogens and risk factors for post-replantation wound infection in patients with traumatic major limb mutilation

**DOI:** 10.1371/journal.pone.0301353

**Published:** 2024-04-01

**Authors:** Chang Gao, Haiyan Wang, Jihui Ju, Keran Zhang, Ye Gao, Shiqi Guo, Di Yin, Ruixing Hou, Qiang Guo

**Affiliations:** 1 Department of Emergency and Critical Care Medicine, The Fourth Affiliated Hospital of Soochow University (Suzhou Dushu Lake Hospital), Suzhou, Jiangsu, China; 2 Medical Center of Soochow University, Suzhou, Jiangsu, China; 3 Medical College of Soochow University, Suzhou, Jiangsu, China; 4 Department of Orthopaedic, Suzhou Ruihua Orthopaedic Hospital (Suzhou Ruixing Medical Group), Suzhou, Jiangsu, China; 5 Department of Critical Care Medicine, Suzhou Ruihua Orthopaedic Hospital (Suzhou Ruixing Medical Group), Suzhou, Jiangsu, China; 6 Department of Critical Care Medicine, Taicang Affiliated Hospital of Soochow University, Suzhou, Jiangsu, China; 7 The First Affiliated Hospital of Soochow University, Suzhou, Jiangsu, China; Policlinico Universitario A. Gemelli IRCCS - Universita Cattolica del Sacro Cuore Roma, ITALY

## Abstract

**Purpose:**

Even though replantation of limb mutilation is increasing, postoperative wound infection can result in increasing the financial and psychological burden of patients. Here, we sought to explore the distribution of pathogens and identify risk factors for postoperative wound infection to help early identification and managements of high-risk patients.

**Methods:**

Adult inpatients with severed traumatic major limb mutilation who underwent replantation from Suzhou Ruixing Medical Group between November 09, 2014, and September 6, 2022 were included in this retrospective study. Demographic, and clinical characteristics, treatments, and outcomes were collected. Data were used to analyze risk factors for postoperative wound infection.

**Results:**

Among the 249 patients, 185 (74.3%) were males, the median age was 47.0 years old. Postoperative wound infection in 74 (29.7%) patients, of whom 51 (20.5%) had infection with multi-drug resistant bacteria. Ischemia time (OR 1.31, 95% CI 1.13–1.53, P = 0.001), wound contamination (OR 6.01, 95% CI 2.38–15.19, P <0.001), and stress hyperglycemia (OR 23.37, 95% CI 2.30–236.93, P = 0.008) were independent risk factors, while the albumin level after surgery (OR 0.94, 95% CI 0.89–0.99, P = 0.031) was significant associated with the decrease of postoperative wound infection. Ischemia time (OR 1.21, 95% CI 1.05–1.40, P = 0.010), wound contamination (OR 8.63, 95% CI 2.91–25.57, P <0.001), and MESS (OR 1.32, 95% CI 1.02–1.71, P = 0.037 were independent risk factors for multi-drug resistant bacteria infection.

**Conclusions:**

Post-replantation wound infection was common in patients with severe traumatic major limb mutilation, and most were multi-drug resistant bacteria. Ischemia time and wound contamination were associated with the increase of postoperative wound infection, including caused by multi-drug resistant. Positive correction of hypoproteinemia and control of stress hyperglycemia may be beneficial.

## Introduction

High energy trauma such as traumatic mutilation of major limbs was the leading cause of amputation and can be life-threatening [1–3]. With the development of surgical techniques, the limbs saving rate of patients gradually increased [1, 4], however, with the aggravation of trauma severity, the incidence of postoperative complications also gradually increased [2, 5, 6]. Postoperative wound infection is one of the most common complications after surgery [7, 8]. Studies showed that Gustilo-Anderson (GA) Classification type III was positively associated with the increased of infection and other complications [8, 9]. Most of the major traumatic limb mutilations were GA type III, nevertheless, the incidence of wound infection after limb salvage surgery was limited.

In patients infected after traumatic fractures, reported strains include but are not limited to Aeromonas, Enterobacter Pseudomonas Enterococcus, Staphylococcus Salmonella, Cutibacterium Proteus, Coagulase negative Staphylococci, and others [10, 11]. The distribution of pathogenic microorganisms varies according to the center, the type of surgery, and even the season [12]. Prophylactic anti-infective measures for GA type III fractures are recommended to cover both gram-negative and gram-positive bacteria, but evidence is limited [13, 14]. With the spread of drug-resistant bacteria in communities and hospitals [15], for patients after replantation, relevant epidemiological characteristics are urgently needed to provide a basis for targeted anti-infection.

In addition, wound infection after replantation can lead to limb loss and increase the psychological and economic burden of patients, with serious adverse consequences [2, 16]. Risk factors for surgical site infection included laboratory findings, trauma grade, and obesity [5, 17, 18], the causes of infection after major limb replantation are poorly understood. Identifying the underlying causes of infections, especially multidrug-resistant bacteria, and intervening early may benefit more patients. Here we sought to identify risk factors for postoperative wound infection to help early identification of high-risk patients.

## Methods

This multicenter retrospective cohort study included all adult patients (age ≥18 years) who had traumatic mutilation of major limbs (defined as an amputation between the trunk and the wrist or ankle) and underwent replantation [19, 20] between November 09, 2014, and September 6, 2022 from the hospitals in the Suzhou Ruixing Medical Group (Suzhou Ruihua Orthopaedic Hospital and Ruixing Hospital). Data were accessed for research purposes on May 1, 2021, data on patients admitted after this date were still collected retrospectively. Ruixing medical group includes Level III specialized hospitals, rehabilitation hospitals, and an institute of applied technology in hand surgery [21]. Orthopedic trauma, amputated limbs (fingers and toes) replantation, and rehabilitation are the focus medical programs of the group, with an average annual operation volume of more than 10,000, which increasing yearly. All of the mutilation limbs were accompanied by discontinuous vessels, nerves, muscles, and bone structures to varying degrees. Patients with severe limb damage that could not be replanted or had first-stage amputations were excluded, which were detailed in our previous study [21]. Identify patients who receive reimplantation by reviewing and analyzing admission logs and histories from all available electronic medical records and patient care resources.

Medical records were reviewed, entered and verified independently by two trained physicians, blinded to each other. Demographic and clinical characteristics including traumatic conditions, laboratory findings, MESS, treatments, and outcomes of the patients were collected. Examinations and treatments during hospitalization were performed by clinicians according to the conditions. Following-up of the patients was from admission to hospital discharge. The primary outcome was the postoperative wound infection rate during hospitalization.

Indications for replantation of severed limb: (1) Relatively complete distal limb and mild skin contusion, (2) The tissue structure of the proximal limb is relatively complete, and the bone and joint injury does not seriously affect the appearance and function of the limb, (3) No avulsive nerve injury or only minor local contusion, (4) Patients could tolerate microsurgery with stable physical signs and without serious complications[21]. Gustilo-Anderson classification of open fractures were defined as follows [22]: type I, open fracture with a wound less than 1 cm long, low energy, without gross contamination; type II, open fracture with a wound 1–10 cm long, low energy, without gross contamination or extensive soft-tissue damage, flaps, or avulsions; type III A, open fracture with a wound greater than 10 cm with adequate soft-tissue coverage, or any open fracture due to high-energy trauma or with gross contamination, regardless of the size of the wound; type III B, open fracture with extensive soft-tissue injury or loss, with periosteal stripping and bone exposure that requires soft-tissue coverage in the form of muscle rotation or transfer; type III C, open fracture associated with arterial injury requiring repair. Stress hyperglycemia was defined as fasting glucose >6.9 mmol/L or random glucose >11.1 mmol/L without evidence of previous diabetes, and preexisting diabetes with deterioration of pre-existing glycemic control [23]. Postoperative wound infection was defined as purulent discharge, erythema, and/or surgical wound dehiscence exposing underlying hardware following definitive fixation and wound closure, necessitating a return to the operating theatre for irrigation and debridement [24]. During surgical irrigation and debridement, at least two separate deep tissue/implant specimens must be collected, returning a phenotypically indistinguishable pathogen, following the consensus statement of an international expert group [24]. Multidrug-resistant was defined as acquired non-susceptibility to at least one agent in three or more antimicrobial categories [25].

The secondary outcomes were the length of ICU and hospital stay, partial/total necrosis, and delayed amputations. Delayed amputations were defined as amputations performed within the same hospitalization period after replantation [26]. Frequency data were expressed as proportions. Continuous data are presented as median (interquartile range [IQR]) if they showed skewed distribution. Shapiro-wilk test was used to determine normal/skewed distribution of the data. Differences in categorical variables were assessed using the χ^2^ test, while comparisons of continuous variables were made using the Mann-Whitney U test, as appropriate. According to whether the data were normally distributed, correlation analysis was performed using Pearson or Spearman, respectively.

To determine the independent risk factors for postoperative wound infection, multivariate logistic regression models were used. Results of the logistic regression were presented as (odds ratio [OR], 95% confidence interval [CI]). Variables with P <0.1 in univariate logistic regression were included in the multivariate analysis. The probabilities of entering and removing variables in a stepwise manner in the multivariate model were 0.05 and 0.10, respectively.

SPSS (version 25.0; IBM, Chicago, IL, USA) was used to analysis data. GraphPad Prism 7 (GraphPad Software, San Diego, CA, USA) and StataMP 16 (StataCorp, College Station, Texas, USA) were used to generate the statistical charts. A two-tailed P value of <0.05 was considered statistically significant.

This study was approved by the Institutional Review Boards of the Suzhou Ruixing Medical Group (2021023). Due to the retrospective nature of the study, no informed consent was required.

## Results

A total of 283 patients were admitted to the hospital after experiencing traumatic major limb mutilation during eight years study period. 34 patients who had either severe limb damage that could not be replanted or first-stage amputations were excluded. 249 patients who underwent replantation were included in this study. The median age of the patients was 47.0 (IQR, 36.0–54.0) years and the majority were males (n = 185, 74.3%). Most patients experienced moderate-to-severe contamination, 91 (36.5%) had lower limb trauma, 181 (72.7%) had blunt trauma, and 100 (40.2%) had total mutilation. The median MESS was 10.0 (IQR, 8.5–12.0) and median ischemia time was 7.5 (IQR, 5.7–9.6) hours. The upper limb salvage rate was 97.5% (154 in 158 cases), the lower limb salvage rate was 95.6% (87 in 91 cases). According to the Gustilo-Anderson Classification for open fractures, all the injuries were type IIIC. All patients received prophylactic antibiotics. The severed part of 249 patients was shown in S1 Table.

### Pathogenic characteristics

**Fig 1:**
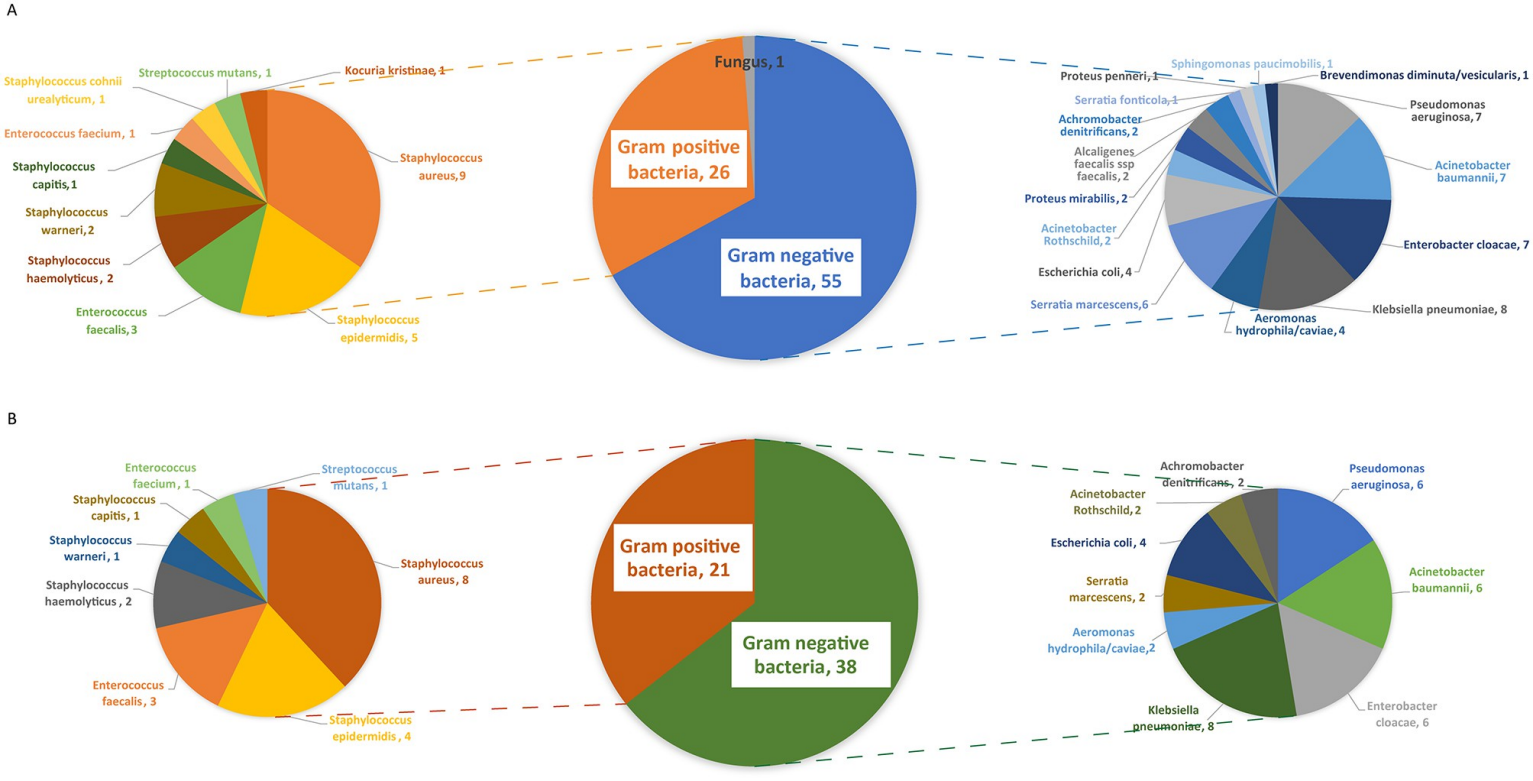
Pathogenic species and distribution of post-replantation wound infection patients. A. Pathogenic species and distribution in 74 postoperative wound infection patients. B. Pathogenic species and distribution in 51 postoperative wound drug-resistant bacterial infection patients.

**Fig 2:**
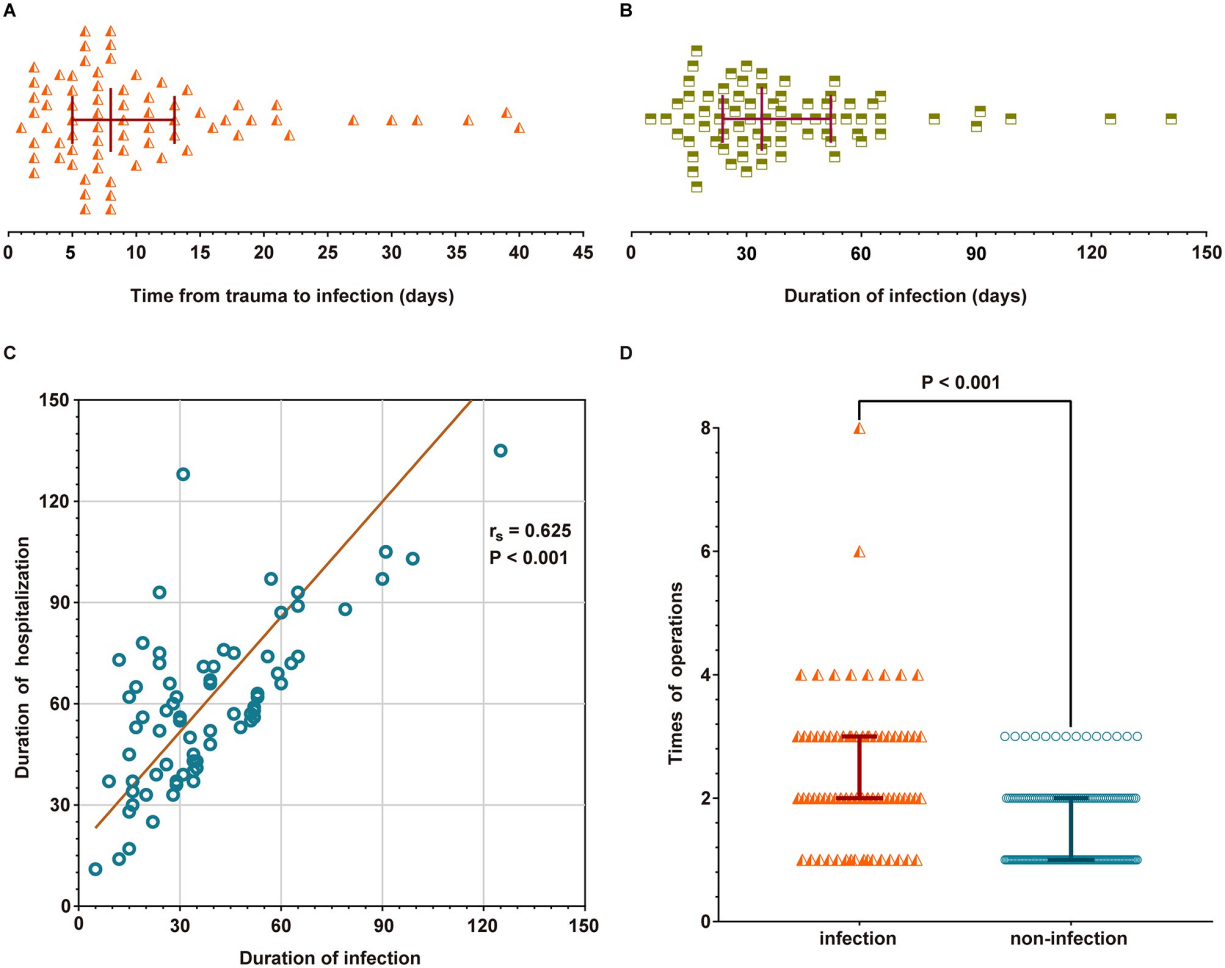
Postoperative wound infection characteristics. A. Time from trauma to infection. B. Duration of postoperative wound infection. C. Linear regression analysis of duration of infection and length of hospital stay. D. Comparison of operation times between infected group and non-infected group.

Postoperative wound infection in 74 (29.7%) patients, of whom 51 (20.5%) had infection with multi-drug resistant bacteria. Of the 74 patients with postoperative wound infection, 45 had only gram-negative bacterial infection, 23 had only Gram-positive bacterial infection, and 6 had mixed infection (Table 1). A total of 82 pathogens were detected, including 26 Gram-positive (G^+^) bacteria, 55 Gram-negative (G^-^) bacteria, and 1 fungus. The top three Gram-positive bacteria were: Staphylococcus aureus, Staphylococcus epidermidis, and Enterococcus; the top three Gram-negative bacteria were: Klebsiella pneumoniae, Enterobacter cloacae, Acinetobacter baumannii, and Pseudomonas aeruginosa (Fig 1A). Among the 82 pathogens, 59 were multi-drug resistant bacteria, including 21 Gram-positive bacteria and 38 Gram-negative bacteria. Among the resistant bacteria, the top three Gram-positive bacteria and the top three Gram-negative bacteria were the same as in all pathogens (Fig 1B). The median time from trauma to infection was 8.0 (IQR, 5.0–13.0) days, median duration of infection was 34.0 (IQR, 23.8–52.0) days (Fig 2A and 2B).

**Table 1:** Pathogenic species of infection.

Pathogenic species of infection	Number of patients
**Gram-negative bacteria (G** ^**-**^ **) only**	**45**
**Gram-positive bacteria (G** ^**+**^ **) only**	**23**
**Mixed infection**	**6**
G^-^ + G^+^	2
G^-^ + G^-^	2
G^-^ + G^-^ + fungus	1
G^+^ + G^-^ + G^-^	1

### Clinical characteristics

Compare with the non-infection group, patients with postoperative wound infection presented with a higher rate of stress hyperglycemia after surgery (9.5% vs. 0.6%, P <0.001), partial/total necrosis (91.9% vs. 50.9%, P <0.001), and delayed amputations (8.1% vs. 1.1%, P = 0.014) (Table 2). The infection group presented higher median values of MESS (12.0 [IQR, 9.0–12.0] vs. 10.0 [IQR, 8.0–11.0], P <0.001), ischemia time (9.0 [IQR, 7.1–11.7] vs. 6.7 [IQR, 5.3–8.5] hours, P <0.001), length of ICU stays (5.0 [IQR, 0.0–8.3] vs. 2.0 [IQR, 0.0–5.0] days, P = 0.002), and length of hospital stays (57.5 [IQR, 41.8–73.3] vs. 36.0 [IQR, 22.0–54.0] days, P <0.001) (Table 2). Patients in postoperative wound infection group had lower median values of red blood cell (RBC) count (3.0 [IQR, 2.3–3.8] vs. 3.3 [IQR, 2.8–3.9] × 10^12^/L, P = 0.027) and platelet count (118.0 [IQR, 69.0–170.0] vs. 147.5 [IQR, 95.0–219.3] × 10^9^/L, P = 0.005) measured immediately after surgery (Table 2).

**Table 2:** Characteristics of 249 patients with replantation of severed limb.

Characteristics	All patients	Infection	Non-infection	*P*
	(n = 249)	(n = 74)	(n = 175)	
Age, median (IQR), yr	47.0 (36.0–54.0)	50.0 (40.0–57.0)	46.0 (32.0–53.0)	0.030
Sex, male patients, n (%)	185 (74.3)	58 (78.4)	127 (72.6)	0.338
Current smokers, n (%)	35 (14.1)	7 (9.5)	28 (16.0)	0.175
Alcohol preference, n (%)	5 (2.0)	1 (1.4)	4 (2.3)	0.631^b^
Pre-existing hypertension, n (%)	17 (6.8)	6 (8.1)	11 (6.3)	0.602
Pre-existing diabetes, n (%)	16 (6.4)	8 (10.8)	8 (4.6)	0.067
Pre-existing cardiovascular disease, n (%)	8 (3.2)	0 (0.0)	8 (4.6)	0.062^*c*^
Heart rate, median (IQR), beats per minute	80.0 (76.0–89.0)	80.0 (77.5–89.0)	80.0 (76.0–89.0)	0.889
Traumatic condition, n (%)				
Lower limb	91 (36.5)	32 (43.2)	59 (33.7)	0.154
Blunt mutilation	181 (72.7)	59 (79.7)	122 (69.7)	0.105
Total mutilation	100 (40.2)	37 (50.0)	63 (36.0)	0.039
5, n (%)				<0.001
Mild	1 (0.4)	0 (0.0)	1 (0.6)	
Moderate	113 (45.4)	15 (20.3)	98 (56.0)	
Severe	135 (54.2)	59 (79.7)	76 (43.4)	
Time from trauma to admission, median (IQR), hr.	2.0 (1.0–3.0)	1.3 (1.0–3.5)	2.0 (1.0–3.0)	0.152
Time from trauma to operation started, median (IQR), hr.	3.3 (2.5–4.5)	3.0 (2.3–4.7)	3.4 (2.5–4.5)	0.458
MESS, median (IQR)	10.0 (8.5–12.0)	12.0 (9.0–12.0)	10.0 (8.0–11.0)	<0.001
Duration of operation, median (IQR), hr.	5.5 (3.5–7.5)	5.9 (3.5–7.8)	5.1 (3.5–7.5)	0.447
Ischemia time, median (IQR), hr. ^a^	7.5 (5.7–9.6)	9.0 (7.1–11.7)	6.7 (5.3–8.5)	<0.001
First laboratory findings after surgery, median (IQR)				
WBC count, × 10^9^/L	10.3 (8.1–12.7)	10.0 (8.3–12.4)	10.3 (8.0–12.9)	0.992
Platelet count, × 10^9^/L	138.0	118.0	147.5	0.005
	(93.0–204.0)	(69.0–170.0)	(95.0–219.3)	
RBC count, × 10^12^/L	3.2 (2.6–3.8)	3.0 (2.3–3.8)	3.3 (2.8–3.9)	0.027
Albumin, g/L	32.1 (26.4–36.1)	31.4 (23.5–35.8)	32.4 (28.3–36.2)	0.078
ALT, U/L	19.3 (13.6–28.1)	18.0 (13.0–29.3)	19.5 (13.6–27.9)	0.965
TBiL, μmol/L	15.0 (9.8–20.0)	15.1 (10.6–20.2)	15.0 (9.4–19.7)	0.521
BUN, mmol/L	4.4 (3.6–5.7)	4.5 (3.8–6.0)	4.4 (3.4–5.2)	0.064
Creatinine, μmol/L	60.9 (52.1–71.1)	61.1 (51.9–75.2)	60.7 (52.3–69.8)	0.369
D-dimer, μg/ml	1.0 (0.4–2.2)	1.2 (0.4–3.4)	0.9 (0.4–2.0)	0.149
Stress hyperglycemia after surgery, n (%)	8 (3.2)	7 (9.5)	1 (0.6)	<0.001^b^
Treatment after surgery, n (%)				
ICU admission	136 (54.6)	45 (60.8)	91 (52.0)	0.202
Anticoagulant therapy	156 (62.7)	53 (71.6)	103 (58.9)	0.057
Antiplatelet therapy	211 (84.7)	65 (87.8)	146 (83.4)	0.377
Outcomes				
Length of ICU stay, day, median (IQR)	2.0 (0.0–6.0)	5.0 (0.0–8.3)	2.0 (0.0–5.0)	0.002
Length of hospital stay, day, median (IQR)	43.0 (27.0–59.5)	57.5 (41.8–73.3)	36.0 (22.0–54.0)	<0.001
Partial/total necrosis, n (%)	157 (63.1)	68 (91.9)	89 (50.9)	<0.001
Delayed amputations, n (%)	8 (3.2)	6 (8.1)	2 (1.1)	0.014 ^b^

^*a*^ Ischemia time was defined as the time from mutilation to recovery of blood circulation.

^*b*^ Yates’s correction was used.

Abbreviations: IQR, interquartile range; WBC, white blood cell; RBC, red blood cell; ALT, alanine aminotransferase; TBiL, total bilirubin; BUN, blood urea nitrogen; MESS, mangled extremity severity score; PT, prothrombin time; ICU, intensive care unit.

Furthermore, in a correlation analysis, we found that the duration of infection was significantly associated with the length of hospital stay (r_s_ = 0.625, P <0.001) (Fig 2C). Patients who suffered postoperative wound infection needed more times of operations than non-infection ones (2.0 [IQR, 2.0–3.0] vs. 1.0 [IQR, 1.0–2.0], P <0.001) (Fig 2D).

### Risk factors for postoperative wound infection and multi-drug resistant bacteria infection

**Fig 3:**
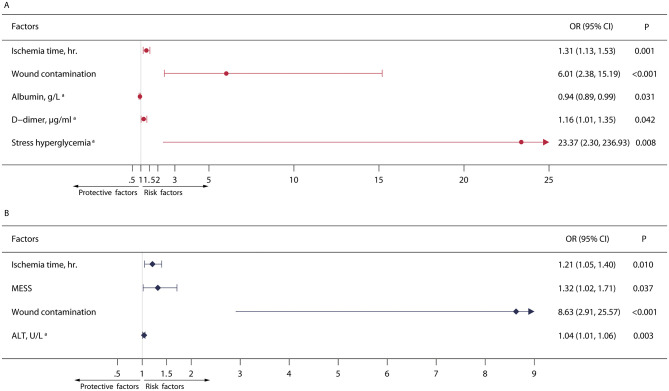
Multivariate logistic analysis of factors associated with post-replantation wound infection. A. Multivariate logistic analysis of factors associated with post-replantation wound infection. B. Multivariate logistic analysis of factors associated with post-replantation wound drug-resistant bacterial infection. ^a^ Conditions or first laboratory findings after surgery.

Univariate logistic regression analysis showed that age, wound contamination, ischemia time, MESS, lactic acid on admission, RBC count immediately after surgery, platelet count after surgery, albumin level after surgery, D-dimer level after surgery, and stress hyperglycemia were significantly associated with Postoperative wound infection (S2 Table). Multivariable logistic regression analysis found that ischemia time (OR 1.31, 95% CI 1.13–1.53, P = 0.001), wound contamination (OR 6.01, 95% CI 2.38–15.19, P <0.001), D-dimer after surgery (OR 1.16, 95% CI 1.01–1.35, P = 0.042), and stress hyperglycemia (OR 23.37, 95% CI 2.30–236.93, P = 0.008) were independent risk factors, while the albumin level after surgery (OR 0.94, 95% CI 0.89–0.99, P = 0.031) was significant associated with the decrease of postoperative wound infection (Fig 3A).

In addition, through univariate logistic regression analysis, results showed that wound contamination, ischemia time, MESS, lactic acid on admission, platelet count after surgery, albumin level after surgery, ALT level after surgery, and D-dimer level after surgery were significantly associated with multi-drug resistant bacteria infection (S3 Table). By multivariable logistic regression analysis, ischemia time (OR 1.21, 95% CI 1.05–1.40, P = 0.010), wound contamination (OR 8.63, 95% CI 2.91–25.57, P <0.001), MESS (OR 1.32, 95% CI 1.02–1.71, P = 0.037), and ALT level after surgery (OR 1.04, 95% CI 1.01–1.06, P = 0.003) were independent risk factors (Fig 3B).

## Discussion

Traumatic major limb mutilation is one of the main causes of limb loss. Even though replantation of limb mutilation is increasing gradually with the development of microsurgery and surgical techniques [1, 4, 27], postoperative wound infection can result in multiple operations and even delayed amputation, which may increase the financial and psychological burden of patients [2, 4, 16]. This study found that postoperative wound infection was common in patients with severe traumatic major limb mutilation, and most of the pathogenic bacteria were multi-drug resistant bacteria, which was associated with ischemic time, degrees of wound contamination, stress hyperglycemia, and MESS.

Surgical site infections, the most common health care-acquired infections, are associated with the emergence of postoperative infected with multidrug-resistant bacteria [7, 28–32]. Previous studies reported that the postoperative wound infection rate of GA classification type III can be even as high as 50% or more [8, 11, 33], which is itself associated with higher postoperative wound infection of fractures [17]. The high incidence of postoperative wound infection in our research may be explained by the fact that the patients are all GA IIIC and had high damage contamination, which leaded to a greater risk of multi-microbial infection [34]. The median time for postoperative wound infection was 8.0 days after trauma. Consistent with previous reports, a strong positive correlation was shown between the duration of the infection and the length of hospital stay, and the number of operations in patients with infection increased significantly, which may lead to an increased chance of acquiring nosocomial infections [35, 36]. Special attention should be paid to the healing or infection status of the wound during this duration after surgery.

The etiological distribution of postoperative wound infection varies according to different surgical reasons, sites and even seasons [9, 11, 12, 37]. Reports on etiological characteristics after regrafting of severe traumatic mutilation of major limbs were limited. For patients with type III open fractures, gram-negative bacilli should be covered in addition to gram-positive bacilli, for which the evidence of evidence-based medicine was insufficient [7, 13, 14, 22]. We here found the main pathogen distribution characteristics, which may provide targeted basis for the use of prophylactic antibiotics in patients in these regional centers.

This study showed that with the increase of wound contamination degree and the prolongation of ischemia time, the incidence of multidrug-resistant bacterial infection increased significantly. The fracture types included in this study were all IIIc types, with large soft tissue, severe vascular injury, and a wide range of exposure, which had high probability of contact with pathogens. Contamination levels and postoperative deep infections have been demonstrated [38], as well as the transmission of multidrug-resistant in communities has become increasingly common [15], which partly explained the high rate of multidrug resistance. Severe open limb trauma had very high risks of tissue ischemia, necrosis and infection. The invasion of pathogenic microorganisms into the damaged skin barrier is easy to cause infection. Tissue ischemia weakens the body’s ability to clear microorganisms, and the necrotic tissue becomes a hotbed for nourishing microorganisms and accelerates the occurrence and development of infection [39]. Therefore, shortening the ischemic time is particularly important for preventing postoperative wound infection [40].

Laboratory markers such as serum albumin [41] and fasting blood glucose [42] were independent risk factors for surgical site infection [18]. In this study, the first postoperative albumin level was collected, which was associated with multi-drug-resistant bacterial infection. Albumin has anti-inflammatory and anti-apoptotic effects [43, 44], and active correction of perioperative hypoproteinemia may be beneficial. Postoperative stress hyperglycemia, not diabetes, was found as a risk factor of infection here. Hyperglycemia can lead to endothelial dysfunction [45], platelet aggregation and thrombosis [46]. A recent study [47] showed that glycemia on arrival in the emergency room was significantly higher in patients with surgical site infection within 1 year after open leg fractures surgery than in patients without infection, and was an independent risk factor for infection. Almost all guidelines recommend the prevention of hyperglycemia to prevent surgical site infections [29, 48]. Treatment of stress hyperglycemia is necessary throughout the treatment course, including in the emergency room and postoperative period. In addition, other scores and laboratory indicators may be used as early warning indicators to help early detection of postoperative wound infection, especially in patients at high risk of multi-drug resistant infection.

Due to the limitations of retrospective study, this study could not fully obtain more detailed details of post-traumatic infection, and did not conduct subgroup analysis of upper and lower limbs or deep and superficial soft tissue infections respectively. It is not possible to explore the resistance genes of multidrug-resistant bacteria and whether there is a synergistic effect when pathogens are co-infected. The specific details of antimicrobial use in all patients were not fully available in the risk factor analysis, and thus could not be used to adjust the risk factors for infection, however, the obtained bacterial distribution characteristics may provide some reference value for future prophylactic antimicrobial use programs. Prospective studies with larger sample sizes and more details of perioperative care are needed to identify potential risk factors.

## Conclusion

Wound infection was common in patients with severe traumatic major limb mutilation after replantation, Gram-negative bacteria were the main pathogenic bacteria, and most of the pathogenic bacteria were multi-drug resistant, which need to be focused on. Ischemia time and wound contamination were associated with the increase of postoperative wound infection, including caused by multi-drug resistant. Positive correction of hypoproteinemia and control of stress hyperglycemia may be beneficial.

## Supporting information

S1 Table The severed part of 249 patients with replantation of severed limb. (DOCX)

S2 Table Univariate logistic analysis of factors associated with postoperative wound infection. (DOCX)

S3 Table Univariate logistic analysis of factors associated with postoperative wound drug-resistant bacterial infection. (DOCX)
