# Supplementary material for: Distribution of pathogens and risk factors for post-replantation wound infection in patients with traumatic major limb mutilation
Source: PLoS One. 2024 Apr 1;19(4):e0301353. doi: 10.1371/journal.pone.0301353 (PMC10984543; doi:10.1371/journal.pone.0301353)
Supplement: S1 Table — (DOCX) [file pone.0301353.s001.docx]

**Supporting information**

**Distribution of pathogens and risk factors for post-replantation wound infection in patients with traumatic major limb** **mutilation**

**S1 Table. The** **severed part of 249 patients with replantation of severed limb.**

| **Severed part of limb** | **Number of patients** |
| --- | --- |
| Forearm | 88 |
| Elbow | 3 |
| Upper arm | 22 |
| Shoulder | 3 |
| Wrist | 33 |
| Wrist + elbow | 1 |
| Forearm + upper arm | 1 |
| Wrist + forearm | 5 |
| Forearm + elbow | 2 |
| Ankle | 58 |
| Shank | 22 |
| Thigh | 9 |
| Shank + ankle | 2 |
