# Supplementary material for: Distribution of pathogens and risk factors for post-replantation wound infection in patients with traumatic major limb mutilation
Source: PLoS One. 2024 Apr 1;19(4):e0301353. doi: 10.1371/journal.pone.0301353 (PMC10984543; doi:10.1371/journal.pone.0301353)
Supplement: S2 Table — (DOCX) [file pone.0301353.s002.docx]

**Supporting information**

**Distribution of pathogens and risk factors for post-replantation wound infection in patients with traumatic major limb** **mutilation**

**S2 Table. Univariate logistic analysis of factors associated with postoperative wound infection.**

| **Factor** | **Unadjusted OR (95% CI)** | **P** |
| --- | --- | --- |
| Age | 1.02 (1.00-1.04) | 0.040 |
| Sex, male patients | 1.37 (0.72-2.61) | 0.339 |
| Current smokers | 0.55 (0.23-1.32) | 0.180 |
| Alcohol preference | 0.59 (0.06-5.33) | 0.635 |
| Pre-existing hypertension | 1.32 (0.47-3.70) | 0.603 |
| Pre-existing diabetes | 2.53 (0.91-7.02) | 0.075 |
| Pre-existing liver disease | 1.19 (0.35-4.09) | 0.779 |
| Heart rate | 1.01 (0.99-1.03) | 0.644 |
| Respiratory rate | 1.01 (0.95-1.07) | 0.868 |
| Lower limb | 1.50 (0.86-2.61) | 0.155 |
| Blunt mutilation | 0.59 (0.31-1.12) | 0.107 |
| Total mutilation | 1.78 (1.03-3.08) | 0.040 |
| Wound contamination | 5.01 (2.69-9.66) | <0.001 |
| Ischemia time | 1.26 (1.15-1.38) | <0.001 |
| MESS | 1.43 (1.22-1.68) | <0.001 |
| WBC count, × 10^9^/L *^a^* | 0.99 (0.96-1.04) | 0.835 |
| Platelet count, × 10^9^/L *^a^* | 0.99 (0.99-0.99) | 0.007 |
| RBC count, × 10^12^/L *^a^* | 0.69 (0.49-0.98) | 0.035 |
| Albumin, g/L *^a^* | 0.96 (0.92-0.99) | 0.023 |
| ALT, U/L *^a^* | 1.01 (0.99-1.03) | 0.131 |
| BUN, mmol/L *^a^* | 1.19 (0.99-1.42) | 0.062 |
| Creatinine, μmol/L *^a^* | 1.01 (0.99-1.03) | 0.103 |
| D-dimer, μg/ml *^a^* | 1.15 (1.02-1.30) | 0.022 |
| Lactate ≥ 4mmol/l on admission | 2.18 (1.05-4.56) | 0.038 |
| Stress hyperglycemia | 18.18 (2.20-150.57) | 0.007 |
| Anticoagulant therapy | 1.76 (0.98-3.18) | 0.059 |
| Antiplatelet therapy | 1.44 (0.64-3.20) | 0.378 |

*^a^* First laboratory findings after surgery.

Abbreviations: IQR, interquartile range; WBC, white blood cell; RBC, red blood cell; ALT, alanine aminotransferase; BUN, blood urea nitrogen; MESS, mangled extremity severity score; PT, prothrombin time; OR, odds ratio.
