# Supplementary material for: Distribution of pathogens and risk factors for post-replantation wound infection in patients with traumatic major limb mutilation
Source: PLoS One. 2024 Apr 1;19(4):e0301353. doi: 10.1371/journal.pone.0301353 (PMC10984543; doi:10.1371/journal.pone.0301353)
Supplement: S3 Table — (DOCX) [file pone.0301353.s003.docx]

**Supporting information**

**Distribution of pathogens and risk factors for post-replantation wound infection in patients with traumatic major limb** **mutilation**

**S3 Table. Univariate logistic analysis of factors associated with postoperative wound drug-resistant bacterial infection.**

| **Factor** | **Unadjusted OR (95% CI)** | **P** |
| --- | --- | --- |
| Age | 1.01 (0.99-1.04) | 0.347 |
| Sex, male patients | 1.01 (0.50-2.05) | 0.969 |
| Current smokers | 0.78 (0.30-1.99) | 0.777 |
| Alcohol preference | 0.98 (0.11-8.87) | 0.978 |
| Pre-existing hypertension | 1.21 (0.38-3.89) | 0.747 |
| Pre-existing diabetes | 2.51 (0.87-7.26) | 0.090 |
| Pre-existing liver disease | 1.31 (0.34-5.04) | 0.692 |
| Heart rate | 1.02 (0.99-1.04) | 0.185 |
| Respiratory rate | 1.02 (0.96-1.08) | 0.563 |
| Lower limb | 0.84 (0.44-1.60) | 0.593 |
| Blunt mutilation | 0.68 (0.33-1.42) | 0.304 |
| Total mutilation | 1.93 (1.04-3.59) | 0.039 |
| Wound contamination | 6.16 (2.76-13.75) | <0.001 |
| Ischemia time | 1.20 (1.10-1.32) | <0.001 |
| MESS | 1.43 (1.19-1.72) | <0.001 |
| WBC count, × 10^9^/L *^a^* | 0.99 (0.95-1.04) | 0.889 |
| Platelet count, × 10^9^/L *^a^* | 0.99 (0.98-0.99) | 0.007 |
| RBC count, × 10^12^/L *^a^* | 0.76 (0.52-1.11) | 0.151 |
| Albumin, g/L *^a^* | 0.95 (0.91-0.99) | 0.025 |
| ALT, U/L *^a^* | 1.02 (1.01-1.04) | 0.009 |
| BUN, mmol/L *^a^* | 1.16 (0.95-1.42) | 0.152 |
| Creatinine, μmol/L *^a^* | 1.01 (0.99-1.03) | 0.154 |
| D-dimer, μg/ml *^a^* | 1.14 (1.00-1.29) | 0.046 |
| Lactate ≥ 4mmol/l on admission | 2.70 (1.23-5.90) | 0.013 |
| Stress hyperglycemia | 4.13 (1.00-17.11) | 0.050 |
| Anticoagulant therapy | 1.98 (0.99-3.95) | 0.052 |
| Antiplatelet therapy | 1.17 (0.48-2.83) | 0.733 |

*^a^* First laboratory findings after surgery.

Abbreviations: IQR, interquartile range; WBC, white blood cell; RBC, red blood cell; ALT, alanine aminotransferase; BUN, blood urea nitrogen; MESS, mangled extremity severity score; PT, prothrombin time; OR, odds ratio.
